# Malaria epidemic and transmission foci in highland of Kisii, western Kenya

**DOI:** 10.1016/j.parepi.2022.e00263

**Published:** 2022-07-20

**Authors:** Kevin O. Ochwedo, Wilfred O. Otambo, Richard R. Olubowa, Isaiah Debrah, Edwin M. Ombima, Ming-Chieh Lee, Richard W. Mukabana, Guiyun Yan, James W. Kazura

**Affiliations:** a Department of Biology, Faculty of Science and Technology, University of Nairobi, Nairobi, Kenya; b Department of Zoology, Maseno University, Kisumu, Kenya; c Department of Biochemistry, Cell and Molecular Biology, West Africa Centre for Cell Biology of Infectious Pathogen, University of Ghana, Accra, Ghana; d Department of Biochemistry and Molecular Biology, Egerton University, Njoro, Kenya; e Program in Public Health, College of Health Sciences, University of California, Irvine, USA; f Centre for Global Health and Diseases, Case Western Reserve University, Cleveland, OH, USA

**Keywords:** *Plasmodium* infections, Epidemic-prone zones, Kisii highland, Microscopic, Submicroscopic

## Abstract

**Background:**

The vulnerable population within the malaria epidemic zone remains at risk of increased burden and fatality. This is because of unpreparedness and overstretching of healthcare capacity in the event of a full-fledged epidemic. The purpose of this study was to determine the prevalence of microscopic and submicroscopic infections, as well as map specific *Plasmodium* transmission foci, in the malaria epidemic-prone zone of Kisii highland.

**Methodology:**

Patients seeking malaria treatment at Eramba health facility in the epidemic-prone zone of Kisii highland were enrolled in the study. Malaria outpatient data for the entire month of May were also included in the analysis. Patients' finger prick blood smears were examined for microscopic infections, while a real-time polymerase chain reaction targeting the *Plasmodium* species 18S rRNA gene was used to detect the presence of submicroscopic infections on DNA extracted from dry blood spots.

**Results:**

Based on outpatient data, the malaria positivity rate was 20.7% (231/1115, 95% CI, 0.18–0.23). The positivity rate varied significantly by age group (χ^2^ = 75.05, df 2, *p* < 0.0001). Children under the age of five had the highest positivity rate (27.8%, 78/281), followed by children aged 5–15 years (19.4%, 69/356), and individuals aged 15 years and above (17.6%, 84/478). Out of the 102 patients recruited, the positivity rate by microscopy was 57.8% (59/102) and 72.5% (74/102) by RT-PCR. Most of the microscopic infections (40.7%, 24/59) were from Morara and Nyabikondo villages in Rioma and Kiomooncha sublocations, respectively. The submicroscopic prevalence was 14.7% (15/102) and was observed only in patients from high-infection villages in Rioma (15.8%, 9/57) and Kiomooncha (16.2%, 6/37) sublocations. Across gender and age groups, females (19.7%, 12/61) and patients aged 15 years and above (21.1%, 8/38) had high levels of submicroscopic infections. There were two mixed infections of *P. falciparum*/*P. malariae* and *P. falciparum*/*P. ovale*, both from patients residing in Kiomooncha sublocation.

**Conclusion:**

*Plasmodium falciparum* infections remained relatively high in the Marani subcounty. Infections were concentrated in two villages, which could serve as a target for future public health intervention, particularly during a malaria epidemic.

## 1 Introduction

Malaria transmission reduction interventions have primarily targeted endemic areas in Kenya, where disease burden and mortality are high. However, epidemic-prone areas, particularly the highlands bordering malaria-endemic areas, remain vulnerable (Ndenga et al., 2006; Wanjala et al., 2011; Wanjala and Kweka, 2016). Because of current changes in rainfall patterns witnessed in the country and rising temperatures, which encourage the breeding of malaria vectors, some epidemic-prone areas in Kenya have recorded prevalence ranging from 10% to 28% (Aidoo et al., 2018; Kapesa et al., 2018; Kipruto et al., 2017). As a result, nearly 20% of Kenyans living in these areas are at risk of Plasmodial infections (President's Malaria Initiative, 2011), and identifying some of the key hotspots in these areas is critical.

Increased deaths have been reported in Rift Valley epidemics-prone counties such as Baringo, Marakwet, and West Pokot as a result of a strained healthcare system during the recent malaria outbreak (Ministry of Health, 2019). For example, in Baringo, 12 villages (including Riong'o, Akwichatis, and Naudo) were severely impacted in 2020, with services in Akwichatis and Riong'o dispensaries being strained. Other recently affected regions include the Bureti subcounty in Kericho (Ministry of Health, 2022). The majority of those affected were children under the age of five and pregnant women. With projected changes in climatic conditions, increased human population, and unpredictability of host activity patterns (Alonso et al., 2011; Krsulovic et al., 2021; Ruiz Carrascal et al., 2014), malaria incidence will continue to rise, particularly in Kenya's highlands bordering endemic zones. To ensure effective planning and monitoring, the Ministry of Health (MoH) will need to map villages with high *Plasmodium falciparum* infection within epidemic zones. The purpose of this study was to determine the prevalence of microscopic and submicroscopic *P. falciparum* infections in clinical patients from the epidemic-prone Kisii highland in western Kenya, with the goal of mapping existing malaria transmission pockets.

## 2 Material and methods

### 2.1 Study area and design

**Fig. 1:**
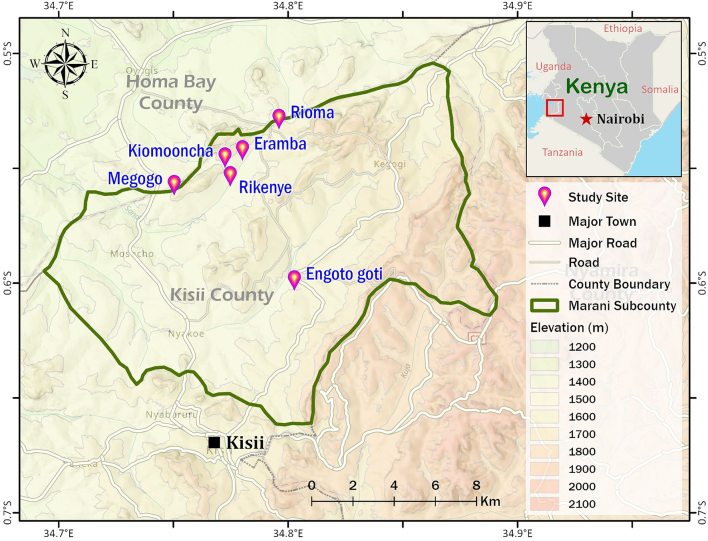
The map depicts the study area (Marani subcounty) in Kisii, as well as health facility and five sublocations from which study participants were traced.

This was a health-based survey conducted at Kisii County's Eramba health facility. The research was conducted in May 2019, when the health facility received 1115 patients seeking malaria treatment. The health facility is located on a highland adjacent to the malaria-endemic Lake Victoria basin in the Marani subcounty, which is administratively divided into locations and sublocations (Fig. 1). Participants in the study were residents of the Engoto Goti, Kiomooncha, Megogo, Rikenye, and Rioma sublocations, which are located at an elevation of 1501–2500 m above sea level (Kweka et al., 2017).

### 2.2 Processing of blood smears

The blood samples from finger prick were spotted on coded Whatman™ Blood Stain Cards (GE Healthcare WB100014) and smears made on glass slides. Microscopists at the hospital prepared and read Giemsa-stained slides for the presence and density of asexual parasites. Microscopy reading, estimation of parasite density and quality control were done as previously described (Ochwedo et al., 2021).

### 2.3 DNA extraction and *Plasmodium* speciation

Parasite DNA was extracted from DBS using the modified Chelex resin (Chelex-100) saponin method (Ochwedo et al., 2021; Onyango et al., 2021; Plowe et al., 1995). The extracted *Plasmodium* DNA was stored at −20 °C. To confirm samples with parasite DNA, a species-specific real-time polymerase chain reaction (RT-PCR) targeting 18 s rRNA was performed. Amplification was carried out on a QuantStudio 3 Real-Time PCR System (ThermoFisher, Carlsbad, CA, USA) with species-specific primers and probes, as previously described (Onyango et al., 2021; Veron et al., 2009). The PCR system's results were exported to an excel spreadsheet for further analysis.

### 2.4 Statistical analysis

Patient data were entered into Microsoft Excel v.2016, and summarized using descriptive statistics. The Pearson chi-square test was used to compare microscopic and submicroscopic *P. falciparum* infections across gender, age groups, and sublocations. The analyses were carried out using SPSS version 25 for Windows and GraphPad Prism v.8.0.1 software. Data were considered statistically significant at *p* < 0.05.

### 2.5 Ethical approval and consenting

Maseno University's Ethical Review Committee granted ethical approval for this study (Reference No. 00456). Participants in the study provided written consent for the retrieval of patient data and the storage of their blood samples at the hospital. Parents or guardians provided consent as children's legal representatives. To avoid ethical code violations, patients' identities were not recorded.

## 3 Results

### 3.1 Positive cases based on outpatient data

Generally, the health facility attracted 1115 patients who sought malaria treatment in the month of May 2019. Females who sought malaria treatment were 78.6% (876/1115) whereas males were 21.4% (239/1115). Across age groups, 42.9% (478/1115) were adults followed by children aged 5–15 years 31.9% (356/1115) and > 5 years 25.2% (281/1115). Based on outpatient data, 935 febrile patients were examined by microscopy and 180 by the CareStart™ malaria HRP2 (Pf) rapid diagnostic kit (mRDT). The hospital's overall positivity rate was 20.7% (231/1115, 95% CI, 0.18–0.23). The positivity rate by microscopy was 20.1% (188/935), while the rate by mRDT was 23.9% (43/180). Despite males having higher proportion of *P. falciparum* infections than females, the difference was not significant (χ^2^ = 2.334, df 1, *p* = 0.127). When compared to adults and children aged 5–15 years, children under the age of five had a higher prevalence of *P. falciparum* infections (Table 1). The variation in malaria positivity rates across age group was significant (χ^2^ = 75.05, df 2, *p* < 0.0001).

**Table 1:** **Malaria positivity rates across gender and age groups**. N represents the total number of individuals while n represents the cases.

Parameter		N (%)	Positive cases n (%)	*P*-Value
Name	Level			
Gender	Female	876 (78.6)	173 (19.7)	0.127
	Male	239 (21.4)	58 (24.3)	
Age group	>5	281 (25.2)	78 (27.8)	<0.0001
	5–15	356 (31.9)	69 (19.4)	
	≥15	478 (42.1)	84 (17.6)	

### 3.2 Characteristics of patients enrolled in the study

Of the 935 outpatients, 102 study participants were randomly recruited. These included participants who were diagnosed by microscopy and their blood spotted on filter paper for RT-PCR assay. Most 57 (55.9%) were from Rioma sublocation whereas the rest were from Kiomooncha 37 (36.2%), Rikenye 6 (5.9%), Engoto Goti 1 (1%) and Megogo 1 (1%) sublocations. Sixty-one (59.8%) were female whereas 41 (40.2%) were males. Children between age group 5–15 years were largest group 41 (40.2%) followed by adults 38 (37.3%) and those <5 years 23 (22.5%). Female dominance was evident across two age groups as follows, 16 (69.6%) and 27 (71.1%) among <5 years and adults (≥15 years) respectively. More than half of recruited participants within the school-going age group (5–15 years) were males 23 (56.1%).

### 3.3 Prevalence of submicroscopic infections among the recruited study participants

A total of 59 (57.8%) of screened blood smears tested positive for microscopic infections. The majority of patients with positive smears 62.7% (37/59) and 27.1% (16/59) were Rioma and Kiomooncha residents, respectively. In the two sublocations, two villages, Morara in Rioma and Nyabikondo in Kiomooncha each had 12 patients with microscopic infections and contributed to 40.8% (24/59) of the slide positive rate. There were 17.6% (15/102) of submicroscopic infections, with female patients infected more than males (Table 2). These infections were beyond the microscopy detection threshold and were slide negative. The difference in submicroscopic infections between the gender was however not significant (χ^2^ = 2.98, df 1, *p* = 0.084). Adults had the most submicroscopic infections 21.1% (8/38) of any age group. The difference across the age groups was however not significant (χ^2^ = 2.072, df 2, *p* = 0.355). These infections were only confirmed in patients from Rioma and Kiomooncha sublocations (Table 2). Two participants from Kengambi village in the Kiomooncha sublocation had mixed infections. One participant had *P. falciparum* and *P. malariae* infections, while the other had *P. falciparum* and *P. ovale* infections. There was no statistically significant difference in submicroscopic *P. falciparum* infections between the five sublocations (χ^2^ = 1.500, df 4, *p* = 0.827). Rioma sublocation had the highest proportion of patients with total infections (microscopic and submicroscopic) (80.7%), followed by Kiomooncha (59.5%), and Rikenye (66.7%). Females had 68.9% more total infections than males (Table 2). Both infection levels were higher in children aged 5–15 years (85.4%), followed by children <5 years (65.2%) and adults (63.2%). However, the observed differences in total infections between gender, age groups, and sublocations were not statistically significant.

**Table 2:** **The proportion of microscopic, submicroscopic and total or all *P. falciparum* infections among gender, age group and sublocations**. N represents the total number of individuals while n represents the cases.

Parameter		N (%)	Microscopic infections n (%)	Submicroscopic infections n (%)	Total infections
Name	Level				
Gender	Female	61 (59.8)	30 (50.8)	12 (19.7)	42 (68.9)
	Male	41 (40.2)	29 (49.2)	3 (7.3)	32 (78)
Age group	>5	23 (22.5)	12 (20.3)	3 (13)	15 (65.2)
	5–15	41 (40.2)	31 (52.5)	4 (9.8)	35 (85.4)
	≥15	38 (37.3)	16 (27.1)	8 (21.1)	24 (63.2)
Sublocation	Engoto Goti	1 (1)	1 (100)	0	1 (100)
	Kiomooncha	37 (36.2)	16 (43.2)	6 (16.2)	22 (59.5)
	Megogo	1 (1)	1 (100)	0	1 (100)
	Rikenye	6 (5.9)	4 (66.7)	0	4 (66.7)
	Rioma	57 (55.9)	37 (64.9)	9 (15.8)	46 (80.7)

## 4 Discussion

In May, the malaria positivity rate among clinically ill patients was high in the epidemic-prone Marani subcounty. Males and children under the age of five were the most affected groups in this epidemic-prone zone, according to the total number of reported malaria cases in the facility. The infections were zoned to two major sublocations, Rioma and Kiomooncha, with transmission foci traced to Morara and Nyabikondo villages, respectively. The levels of submicroscopic infections, on the other hand, were low and were only confirmed on patients from the two sublocations. Females bore a disproportionate share of the submicroscopic infections compared to males, with high prevalence of total infections and parasite densities still being recorded among children aged 5–15 years.

As observed in endemic zones, more females in Kisii highland sought malaria treatment at health facility as compared to males (Ochwedo et al., 2021). Furthermore, there was no difference in health seeking trends by age group from reports in the endemic region, with more adults seeking treatment, followed by children aged 5–15 and under 5 years, respectively. The observed malaria positivity rate was slightly lower (20.7% vs. 26.7% vs. 27.8%) among previously reported cases by Kapesa et al. (2018) and Aidoo et al. (2018). With the insignificant variation in malaria positivity rates, the study hypothesizes that the average malaria positivity rate for the Marani subcounty could be around 21–28%. This, however, needs to be investigated further by conducting monthly malaria surveillance. Also, there are reports of a slight increase in malaria cases following the COVID-19 pandemic.

The majority of the microscopic infections observed were focal and could be traced back to Rioma or Kiomooncha sublocations. The two sublocations also had the highest number of patients seeking malaria treatment and are adjacent to each other while bordering an endemic region, Homa Bay County. Their proximity to endemic areas may predispose residents to malaria infections (Kenya Ministry of Health, 1994), as well as put the sublocations at high risk of imported *P. falciparum*. Morara and Nyabikondo villages in Rioma and Kiomooncha, which have a high number of microscopic infected individuals, serve as a guide for possible focal parasite or vectorial intervention targets in the event of a future outbreak of *P. falciparum* infections in the Marani subcounty.

Submicroscopic infections were relatively low, with females and adult patients having the highest levels. The slightly higher prevalence of this type of infection in females and adults supports previous findings and has been linked partly to immunity (Ochwedo et al., 2021; Whittaker et al., 2021). However, the difference in submicroscopic infection levels across gender and age groups was not statistically significant, as observed in other health-based surveys in endemic zones (Ochwedo et al., 2021). The similarity of parasite rates across the age spectrum is consistent with the study area being a highland or fringe zone. Individuals with submicroscopic infections were found in Kiomooncha and Rioma sublocations. The general low levels of submicroscopic and high levels of microscopic infections among patients presenting with clinical symptoms in epidemic-prone zones could be attributed to a low rate of clinical immunity buildup (Cook et al., 2019).

## 5 Conclusion

This study established high malaria positivity rates and low levels of submicroscopic infections in five sublocations in the epidemic-prone region of Kisii county. The transmission was focalized to two main sublocation of which two key villages were observed as sentinel sites with the potential targeted parasite or vectorial intervention in case of malaria epidemic.

## Funding sources

The work was supported by Grants from the 10.13039/100000002National Institutes of Health (U19 AI129326 and D43 TW001505).

## Declaration of Competing Interest

Authors have no conflict of interest to disclose.
